# Acidity of Carboxylic Acid Ligands Influences the Formation of VO_2_(A) and VO_2_(B) Nanocrystals under Solvothermal Conditions

**DOI:** 10.1021/acsnanoscienceau.3c00014

**Published:** 2023-06-22

**Authors:** Brittney A. Beidelman, Xiaotian Zhang, Ellen M. Matson, Kathryn E. Knowles

**Affiliations:** Department of Chemistry, University of Rochester, Rochester, New York 14627, United States

**Keywords:** vanadium dioxide, solvothermal synthesis, nanocrystals, precursor conversion, polymorphs

## Abstract

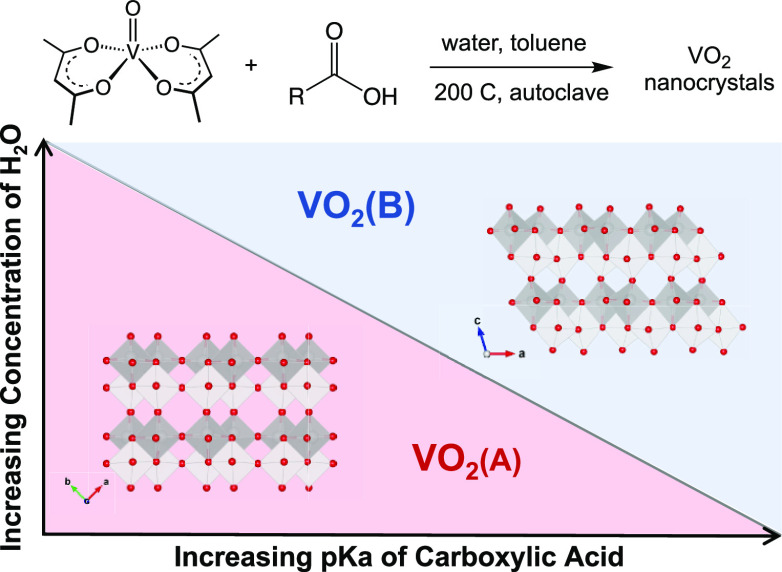

Vanadium dioxide (VO_2_) can adopt many different crystal structures at ambient temperature and pressure, each with different, and often desirable, electronic, optical, and chemical properties. Understanding how to control which crystal phase forms under various reaction conditions is therefore crucial to developing VO_2_ for various applications. This paper describes the impact of ligand acidity on the formation of VO_2_ nanocrystals from the solvothermal reaction of vanadyl acetylacetonate (VO(acac)_2_) with stoichiometric amounts of water. Carboxylic acids examined herein favor the formation of the monoclinic VO_2_(B) phase over the tetragonal VO_2_(A) phase as the concentration of water in the reaction increases. However, the threshold concentration of water required to obtain phase-pure VO_2_(B) nanocrystals increases as the p*K*_a_ of the carboxylic acid decreases. We also observe that increasing the concentration of VO(acac)_2_ or the concentration of acid while keeping the concentration of water constant favors the formation of VO_2_(A). Single-crystal electron diffraction measurements enable the identification of vanadyl carboxylate species formed in reactions that do not contain enough water to promote the formation of VO_2_. Increasing the length of the carbon chain on aliphatic carboxylic acids did not impact the phase of VO_2_ nanocrystals obtained but did result in a change from nanorod to nanoplatelet morphology. These results suggest that inhibiting the rate of hydrolysis of the VO(acac)_2_ precursor either by decreasing the ratio of water to VO(acac)_2_ or by increasing the fraction of water molecules that are protonated favors the formation of VO_2_(A) over VO_2_(B).

## Introduction

Vanadium dioxide (VO_2_) has attracted significant attention because it exhibits multiple crystal phases, all with different properties and uses. The most stable bulk phase, VO_2_(R), with a rutile structure, is well known for its low-temperature metal-to-insulator transition to VO_2_(M) and is used for smart window applications.^1,2^ Like VO_2_(M), the metastable tetragonal phase known as VO_2_(A) has also been investigated as a material for optical switches,^3,4^ while the metastable monoclinic phase known as VO_2_(B) has been used as a cathode material in lithium- and sodium-ion batteries.^5−11^ All of these applications benefit from the solution processability afforded by nanocrystalline morphologies. Given the variation in the application of multiple crystal polymorphs of VO_2_, there is considerable motivation to understand how to control which polymorphs form under various reaction conditions.

Nanocrystals of the metastable polymorphs VO_2_(A) and VO_2_(B) are typically synthesized using solvothermal methods at elevated pressure.^6,8−10,12−18^ These methods often utilize V_2_O_5_ and a reductant in water, and control over the crystal phase is usually achieved by varying the reaction temperature. VO_2_(A) is the major product obtained at reaction temperatures between 220 and 270 °C, and VO_2_(B) is the major product obtained at temperatures between 180 and 200 °C.^6,8−10,12−15^ Varying the pressure by varying the volume of the reaction mixture within the pressure-sealed reaction vessel^19^ also impacts the phase of the resulting product. One study showed that a mixture containing V_2_O_5_ and oxalic acid reacting at 180 °C in a pressure-sealed autoclave could result in phase-pure VO_2_(B) after 24 h and phase-pure VO_2_(A) after 7 days.^20^ However, this result could only be obtained with a filling ratio of 80/100 mL in the pressure-sealed vessel: a filling ratio of 60/100 mL only yielded VO_2_(B) with no phase change present after 7 days.

Recently, we demonstrated that tuning the chemical rather than physical reaction conditions enables control over the crystal phase of VO_2_ nanocrystals. Specifically, we showed that decreasing the concentration of water present in the solvothermal reaction mixture of vanadyl acetylacetonate (VO(acac)_2_) and lauric acid in toluene to 4 equiv or less per vanadium center produces phase-pure VO_2_(A) nanocrystals, whereas using 20 equiv or more of water produces phase-pure VO_2_(B) nanocrystals.^21^ This approach has the added advantage of enabling access to smaller nanocrystals with dimensions less than 500 nm. In contrast, reactions that use water as the solvent produce very large particles, usually nanorods that are several microns long.^6,9,10^ We hypothesize that the mechanism by which the concentration of water impacts the crystal phase of VO_2_ nanocrystals is through controlling the relative rates of precursor hydrolysis and condensation. Decreasing the concentration of water slows hydrolysis and may allow nanocrystal nucleation via condensation of partially hydrolyzed species. We suspect that condensation of these partially hydrolyzed species favors the formation of VO_2_(A) over VO_2_(B).

Here, we test this hypothesis by tuning the p*K*_a_ and steric bulk of organic carboxylic acid ligands to vary the rate of precursor hydrolysis. We find that replacing lauric acid with a stronger acid, namely trifluoroacetic acid, expands the range of water concentrations that lead to the formation of VO_2_(A) nanocrystals by a factor of almost four, up to 15 equiv of water per vanadium center. Increasing the concentration of trifluoroacetic acid and/or the VO(acac)_2_ precursor relative to water also favors the formation of VO_2_(A) over VO_2_(B). These experiments indicate that reaction conditions that suppress the rate of precursor hydrolysis (i.e., decreasing the water concentration or increasing the acidity of the reaction mixture) favor the formation of VO_2_(A).^21^ In contrast to varying the acidity of carboxylic acid, changing the chain length of aliphatic carboxylic acids does not alter the crystal phase of the product. This observation indicates that the steric bulk of these ligands does not alter the relative rates of precursor hydrolysis and condensation significantly enough to impact which crystal phase of VO_2_ nucleates.

## Results and Discussion

**Figure 1:**
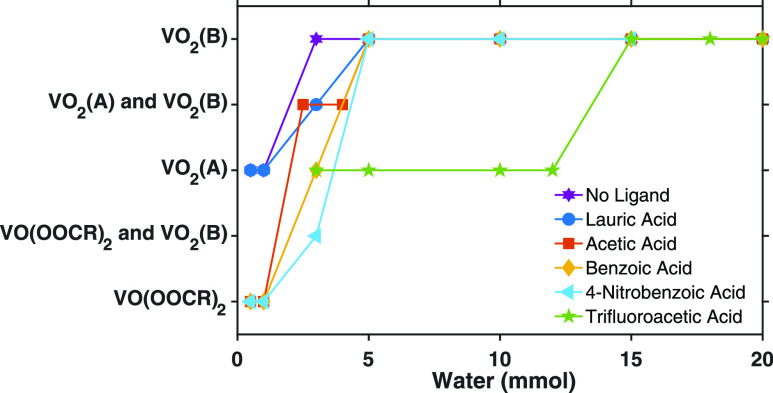
Plot of crystal phases obtained from solvothermal reactions of 0.25 mmol VO(acac)_2_ in toluene with 4 equiv (1 mmol) of carboxylic acid and varying water concentrations (2–100 equiv). Crystal structures were determined using powder X-ray diffraction, and the data can be found in the Supporting Information (Figures S1–S6).

We set out to investigate the role of carboxylic acid ligands in the synthesis of VO_2_ nanocrystals from VO(acac)_2_. We varied two properties of these ligands—their acidity and their steric bulk—to determine which property has the largest influence on the resulting nanocrystals. Our previous work demonstrates that the amount of water present in a solvothermal reaction with VO(acac)_2_ and lauric acid in toluene determines both the crystal phase of VO_2_ synthesized, as well as the length of the resulting nanorods.^21^ Using this synthesis methodology, we varied the amount of water present in the reaction from 0.5 to 20 mmol (2–80 equiv per vanadium) in the presence of 4 equiv (1 mmol) of a selection of acids with varying p*K*_a_’s shown in Table 1. Figure 1 shows that, for each carboxylic acid examined here, a transition occurs from VO_2_(A) products (or product mixtures containing VO_2_(A)) to phase-pure VO_2_(B) products as the amount of water present in the reaction increases past a particular threshold concentration. This observation is consistent with our previously reported study in which the threshold concentration of water required for the formation of phase-pure VO_2_(B) in the presence of lauric acid (p*K*_a_ = 5.3) was determined to be 5 mmol (20 equiv per vanadium).^21^ Here we observe that decreasing the p*K*_a_ of the carboxylic acid increases the threshold concentration of water required to obtain VO_2_(B) instead of VO_2_(A). For example, trifluoroacetic acid (p*K*_a_ = 0.23) has a threshold concentration of 15 mmol (60 equiv per vanadium). Notably, the threshold water concentration observed in the absence of acidic ligands is 3 mmol (12 equiv per vanadium). This observation suggests that the minimum concentration of water required to form phase-pure VO_2_(B) is 12 equiv per vanadium center. Addition of carboxylic acids increases this threshold.

**Table 1:** Aqueous p*K*_a_’s of Carboxylic Acids Used in the Synthesis of VO_2_ Nanocrystals

acid	p*K*_a_	solvent	references
lauric acid	5.3	water	(22)
acetic acid	4.76	water	(22)
benzoic acid	4.20	water	(23)
4-nitrobenzoic acid	3.44	water	(24)
trifluoroacetic acid	0.23	water	(25)

**Figure 2:**
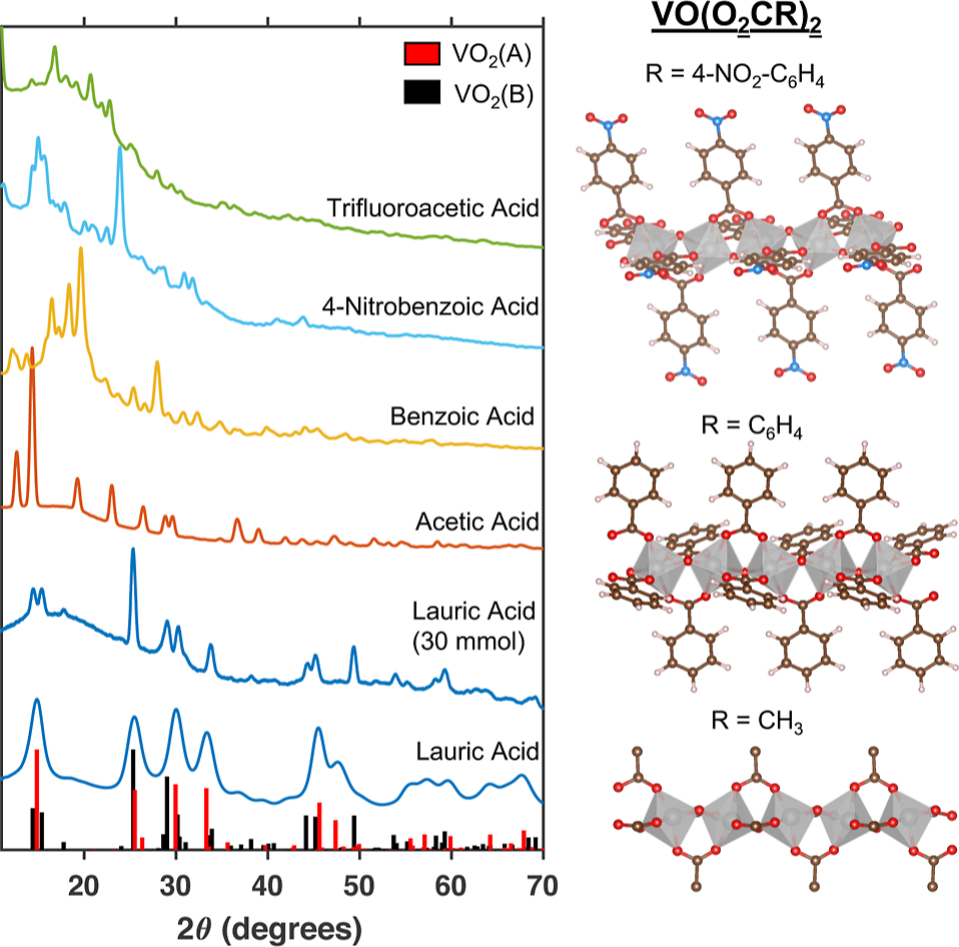
(left) Powder X-ray diffraction spectra of products obtained from solvothermal reactions containing 1 equiv (0.25 mmol) VO(acac)_2_, 4 equiv (1 mmol) acid, 4 equiv (1 mmol) water, and 10 mL of toluene. The powder X-ray diffraction spectrum of the product obtained from a reaction containing 4 equiv of lauric acid and 120 equiv (30 mmol) of water is included for reference as an example of a VO_2_(B) nanocrystal product. Reference powder patterns for VO_2_(A) (JCPDS 00-042-0876, red) and VO_2_(B) (JCPDS 01-081-2392, black) are also included. (right) Crystal structures of the confirmed products include VO(4-nitrobenzoate)_2_ (top), VO(benzoate)_2_ (middle), and VO(acetate)_2_ (bottom).

We also observe that in the presence of very low concentrations of water (1 mmol or less), the addition of acetic, benzoic, 4-nitrobenzoic, and trifluoroacetic acid results in products whose powder X-ray diffraction patterns do not match that of either VO_2_(A) or VO_2_(B) (Figure 2). In the case of acetic acid, this pattern matches that of a previously reported VO(acetate)_2_ crystal structure.^26^ This structure is a coordination polymer comprised of one-dimensional chains of corner-sharing octahedrally coordinated vanadium ions. Each vanadium ion is linked to each of its two neighboring vanadium ions by one bridging oxo ligand and two bridging bidentate acetate ligands. Analysis of single nanocrystals of the products obtained from reactions containing 4 equiv of benzoic or 4-nitrobenzoic acid and 4 equiv (1 mmol) of water by electron diffraction suggests that these products are the corresponding vanadyl carboxylate compounds—VO(benzoate)_2_ and VO(4-nitrobenzoate)_2_—and that these compounds also crystallize as one-dimensional chains of octahedrally coordinated vanadium ions with a structure that is entirely analogous to VO(acetate)_2_ (Figure 2, details of structural characterization in the Supporting Information). The powder X-ray diffraction pattern of the product obtained from the reaction of VO(acac)_2_ with 4 equiv of trifluoroacetic acid and 4 equiv of water resembles that of the other confirmed vanadyl carboxylate species. We therefore strongly suspect that this pattern corresponds to VO(trifluoroacetate)_2_. These data indicate that carboxylic acids can displace acetylacetonate, and 1 mmol (4 equiv) of water is insufficient to hydrolyze these species to form VO_2_. In a solvothermal reaction utilizing VO(benzoate)_2_ as a precursor in the absence of water and benzoic acid, the VO(benzoate)_2_ precursor is recovered. VO(benzoate)_2_ also forms upon reaction of 4 equiv of benzoic acid with VO(acac)_2_ in the absence of water under the same solvothermal conditions (200 °C for 24 h, see Supporting Information). This observation indicates that ligand exchange to form the VO(carboxylate)_2_ species does not require the presence of water.

The earliest reports of coordination polymers of VO(acetate)_2_, VO(benzoate)_2_, and VO(4-nitrobenzoate)_2_ state that these species are insoluble in most solvents, which is consistent with our observations.^27−29^ Although VO(trifluoroacetate)_2_ has been reported previously as a soluble monomeric complex,^30^ the species we isolated from the reaction of VO(acac)_2_ in the presence of 4 equiv each of trifluoroacetic acid and water is not soluble in polar or nonpolar solvents. We therefore suspect that this species is also a coordination polymer. VO(benzoate)_2_ and VO(4-nitrobenzoate)_2_ are reported to crash out immediately upon addition of vanadyl sulfate to an aqueous solution of the corresponding carboxylate,^29^ indicating that monomeric carboxylate complexes cannot be isolated even in the presence of excess acid. Titration of acetic acid into aqueous solutions of the vanadyl ion VO^2+^ achieves a maximum of 1.5 bound acetates per solvated vanadium center,^31,32^ indicating that stable solutions in which all monomeric species are bound to two acetate ligands cannot be achieved. Based on these previous reports, we conclude that monomeric vanadyl acetate, benzoate, or 4-nitrobenzoate species are highly unlikely to exist in nonpolar toluene solutions containing 4 equiv of carboxylic acid per vanadium, such as those used here.

In contrast to benzoic acid, reaction of lauric acid with VO(acac)_2_ in the absence of water produces no reaction—the recovered reaction mixture still contains lauric acid and VO(acac)_2_ (see Supporting Information). Furthermore, we note that formation of VO_2_(A) occurs in the presence of lauric acid and 0.5 or 1 mmol water, whereas at least 2 mmol water (8 equiv) is required to observe the formation of any VO_2_ species in the presence of acetic, benzoic, 4-nitrobenzoic, or trifluoroacetic acid (Figure 1). We suspect that formation of the one-dimensional vanadyl carboxylate coordination polymers and subsequent precipitation of these species impedes hydrolysis of the vanadium centers. We suspect that the steric bulk of the twelve-carbon chain in lauric acid prevents the formation of an analogous VO(laurate)_2_ coordination polymer, thereby leaving the vanadium centers more available for hydrolysis at low water concentrations. Although the powder X-ray diffraction data reported here and the FTIR data we reported previously^21^ indicate that both VO(acac)_2_ and lauric acid are intact in the mixture recovered from a solvothermal reaction run in the absence of water, we cannot completely rule out the formation of some monomeric vanadyl laurate species in solution or the presence of a small fraction of such species in the recovered reaction mixture.

Previous reports have posited that the mechanism for the formation of VO_2_ from the solvothermal reaction of VO(acac)_2_ involves hydrolysis of VO(acac)_2_ to form [VO(H_2_O)_5_]^2+^ followed by condensation of this species to generate VO_2_.^17,18,21^ This vanadyl aquo complex is stable under neutral aqueous conditions^33,34^ and has a p*K*_a_ of 5.3–6.0.^35^ We proposed in our previous work that the relative rate of hydrolysis versus condensation controls whether VO_2_(A) or VO_2_(B) nuclei form. Slow hydrolysis enables condensation of partially hydrolyzed species, whereas faster hydrolysis promotes the formation of the fully hydrolyzed species before significant condensation occurs. We hypothesized that condensation of the fully hydrolyzed species favors the formation of VO_2_(B) nuclei, while condensation of partially hydrolyzed species favors the formation of VO_2_(A) nuclei. Here, we observe that addition of stronger carboxylic acids increases the threshold concentration of water required to obtain phase-pure VO_2_(B) nanocrystals instead of VO_2_(A). This observation is consistent with our hypothesis that the rate of hydrolysis controls the crystal phase of VO_2_ because increasing the strength of the organic acid should inhibit hydrolysis of VO(acac)_2_. More acidic reaction conditions result in a higher ratio of positively charged hydronium ions to water molecules, which lowers the concentration of neutrally charged water available to hydrolyze the VO(acac)_2_. Hydronium ions are much less nucleophilic than neutral water and therefore less reactive toward hydrolysis.

**Figure 3:**
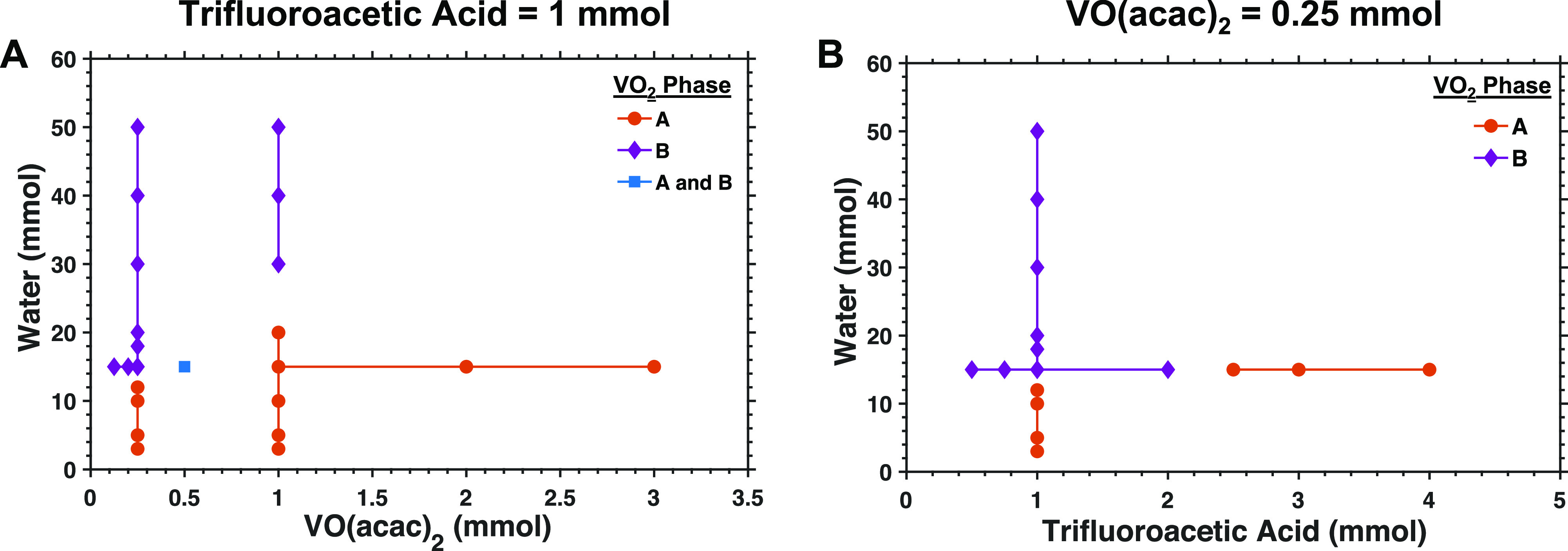
Plots of the crystal phase of VO_2_ nanocrystals obtained from reaction mixtures containing (A) various concentrations of VO(acac)_2_ and water with a constant concentration of trifluoroacetic acid, and (B) various concentrations of trifluoroacetic acid and water with a constant concentration of VO(acac)_2_. The amounts of reagents used in each of these reactions are tabulated in Table S3, and the powder X-ray diffraction spectra used to construct these plots are shown in the Supporting Information (Figures S9–S12).

We further tested our hypothesis that slow hydrolysis promotes the formation of VO_2_(A) over VO_2_(B) by conducting a series of reactions in which we varied the relative concentrations of VO(acac)_2_, water, and trifluoroacetic acid (Figure 3). VO_2_(A) becomes the favored product as the concentration of either VO(acac)_2_ or trifluoroacetic acid increases relative to the concentration of water. Figure 3A shows that, when the amount of trifluoroacetic acid is fixed to 1 mmol, increasing the amount of VO(acac)_2_ from 0.25 to 1 mmol while keeping the concentration of water constant at 15 mmol results in a transition from VO_2_(B) products to VO_2_(A) products. Obtaining VO_2_(B) from a reaction containing 1 mmol of VO(acac)_2_ and 1 mmol trifluoroacetic acid requires increasing the amount of water present to at least 30 mmol, whereas VO_2_(B) can be obtained using only 15 mmol of water in the presence of 0.25 mmol of VO(acac)_2_ and 1 mmol trifluoroacetic acid. Figure 3B shows that similar results are obtained when the amount of VO(acac)_2_ is kept constant at 0.25 mmol and the ratio of trifluoroacetic acid to water is increased by either decreasing the concentration of water or increasing the concentration of acid. In both cases, higher ratios of trifluoroacetic acid to water favor the formation of VO_2_(A).

**Figure 4:**
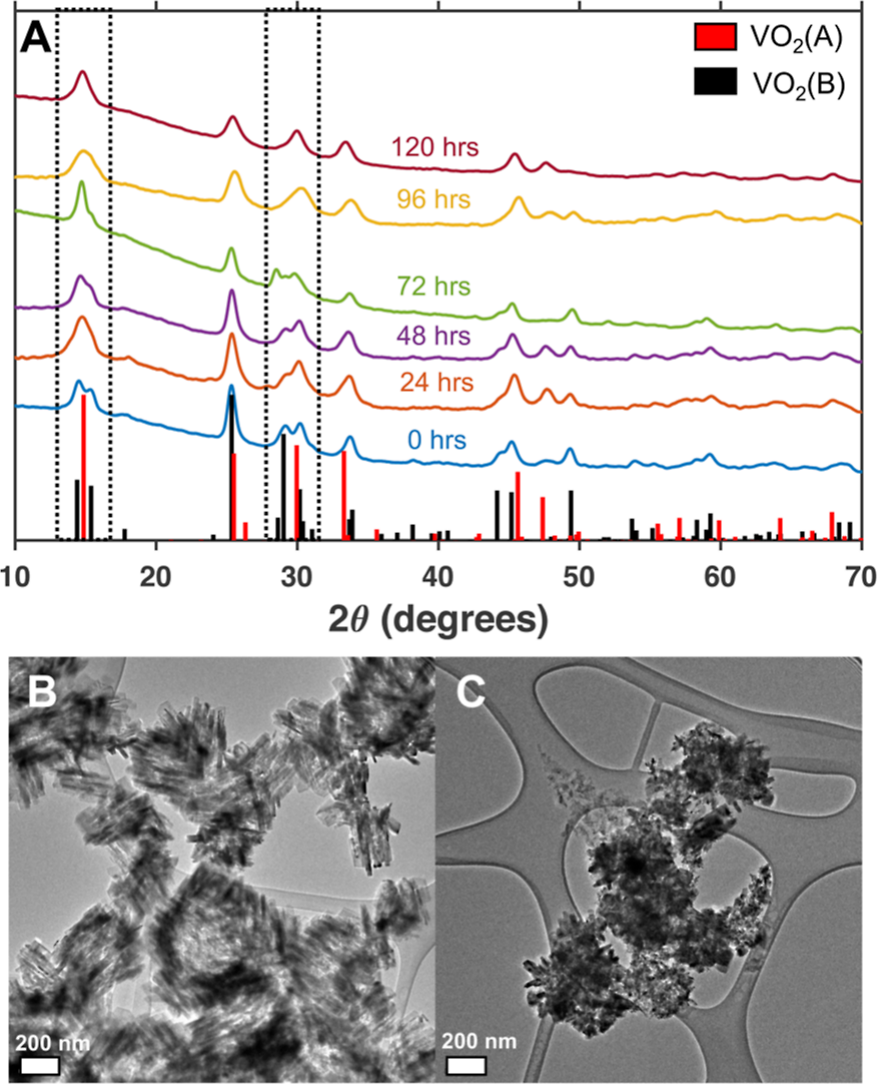
(A) Powder X-ray diffraction spectra of nanocrystals obtained from solvothermal reactions of VO_2_(B) nanocrystals in toluene with 20 equiv of water and 4 equiv of trifluoroacetic acid at 230 °C demonstrate the transformation from VO_2_(B) to VO_2_(A) after 120 h of reaction time. The black dotted rectangles highlight the pairs of diffraction peaks in VO_2_(B) that coalesce to form the peaks associated with diffraction of the (110) and (220) planes of VO_2_(A). (B,C) TEM images of (B) the VO_2_(B) nanocrystals used as the starting material and (C) the VO_2_(A) nanocrystals obtained after reacting the nanocrystals shown in B in toluene with 20 equiv of water and 4 equiv of trifluoroacetic acid at 230 °C for 120 h. The scale bars correspond to 200 nm.

Since the presence of strong carboxylic acids favors the formation of VO_2_(A) and VO_2_(A) is a more thermodynamically stable phase than VO_2_(B), we investigated whether strong carboxylic acids could mediate the conversion of VO_2_(B) nanocrystals to VO_2_(A). Previous reports show that conversion from VO_2_(B) to VO_2_(A) is possible under solvothermal conditions at high temperatures.^3,9^ For example, Zhang, et al. obtained VO_2_(A) nanocrystals upon reacting a solution containing VO_2_(B) nanocrystals and water at 260 or 280 °C for 48 h.^3^ Additionally, another report also heated VO_2_(B) nanocrystals to 280 °C under hydrothermal conditions for 48 h to convert them to VO_2_(A).^9^ We investigated the possibility that lowering the temperature and using an acidic reaction environment could also instigate this transformation. Figure 4 demonstrates that VO_2_(B) nanocrystals can indeed be converted to VO_2_(A) when heated in toluene in the presence of 4 equiv of trifluoroacetic acid and 20 equiv of water at 230 °C over the course of 120 h. This conversion is indicated by the coalescence of two pairs of diffraction peaks at 2θ = 14.4 and 15.3° and 2θ = 29 and 30.2° into single peaks at 2θ = 15 and 30°, respectively. The resulting peaks correspond to the (110) and (220) planes of the VO_2_(A) structure. The overall decrease in the number of diffraction peaks signals an increase in symmetry as the monoclinic VO_2_(B) structure converts to the tetragonal VO_2_(A) structure. The VO_2_(A) nanocrystals obtained from this reaction are smaller than the initial VO_2_(B) nanocrystals (Figure 4). This change in morphology indicates that the trifluoroacetic acid may induce dissolution of the VO_2_(B) nanocrystals and re-nucleation as VO_2_(A), possibly using VO(trifluoroacetate)_2_ as a reaction intermediate. We note that in reactions containing VO(acac)_2_, lauric acid, and 2 or 20 equiv of water, we do not observe any indications of phase interconversion over the course of a 24 h reaction period. Examining products present in these reaction mixtures after various reaction times reveals that only one type of crystalline product is observed per reaction condition: VO_2_(A) for the reaction containing 2 equiv of water and VO_2_(B) for the reaction containing 20 equiv of water (see Supporting Information). This observation implies that for reactions starting from VO(acac)_2_, the final VO_2_ product phase nucleates directly from condensation of hydroxylated species formed upon precursor hydrolysis.

After establishing that the acidity of the carboxylic acid ligands impacts the phase of VO_2_ nanocrystals, we next investigated the impact of tuning the steric bulk of the carboxylic acid ligand on the solvothermal synthesis of VO_2_ nanocrystals. A selection of aliphatic carboxylic acids with varying carbon-chain lengths was chosen, specifically butyric (4C), hexanoic (6C), heptanoic (7C), decanoic (10C), lauric (12C), and stearic (18C) acid. All of these carboxylates have relatively similar p*K*_a_’s of ∼4.8–5.3.^22,36^ Figure S14 in the Supporting Information shows powder X-ray diffraction spectra obtained from reactions of VO(acac)_2_ in the presence of 4 equiv of each of these acids and 20 equiv of water. These data demonstrate that VO_2_(B) is obtained in every case. Likewise, the morphology of the nanocrystals remains constant with variation of the ligand chain length, except for stearic acid, as shown in Figure S14. Reactions containing aliphatic carboxylic acids with chain lengths less than 18 carbons produce VO_2_(B) nanorods of average length 110–150 nm, while stearic acid yields nanoplatelets in addition to nanorods. Overall, since only one of the aliphatic carboxylic acids resulted in a difference in morphology, we conclude that varying the length of aliphatic carboxylic acids does not strongly influence the hydrolysis and growth of VO_2_(B) nanocrystals.

**Figure 5:**
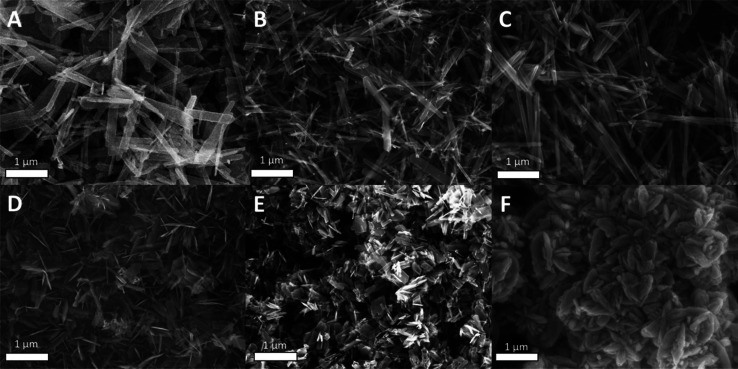
SEM images of reactions with 1 equiv (0.25 mmol) VO(acac)_2_, 60 equiv (15 mmol) water, and 4 equiv (1 mmol) of (A) no ligand, (B) lauric acid, (C) acetic acid, (D) benzoic acid, (E) 4-nitrobenzoic acid, and (F) trifluoroacetic acid. Each image contains a scale bar corresponding to 1 μm.

We also compared the morphologies of VO_2_(B) nanocrystals synthesized with carboxylic acids of various p*K*_a_’s. Since all of the acids produce phase-pure VO_2_(B) with 60 equiv of water, these products were analyzed by scanning electron microscopy (SEM, Figure 5). The reactions with no ligand, lauric acid, and acetic acid all showed nanorod morphologies, while the reactions with benzoic acid and 4-nitrobenzoic acid resulted in a mixture of platelets and nanorods. Although the exact mechanism governing the change in morphology from rods to platelets is unclear, we note that the nanoplatelet morphology affords a larger flat surface area that may promote stabilizing intermolecular interactions between surface-bound ligands, such as π–π interactions between the aromatic acids. We also observe a mixture of nanoplatelet and nanorod products from reactions that contain stearic acid. Long carbon chains are also known to engage in stabilizing intermolecular van der Waal’s interactions on flat surfaces.^37^

Finally, the reaction containing trifluoroacetic acid produced nanocarambolas, which are elongated aggregates of stacked nanosheets whose cross section resembles a six-pointed star.^38^ This morphology was previously observed in the synthesis of VO_2_(B) nanocrystals from VO(acac)_2_ in the presence of polyvinylpyrrolidone (PVP)^18^ and from V_2_O_5_ in the presence of oxalic acid.^7^ The first study discovered that changing the concentration of PVP in the reaction impacted the morphology of the resulting nanocrystals. Nanorods formed in the absence of PVP, while addition of 3 mg/L PVP formed nanoflowers, and 54 mg/L PVP formed nanocarambolas.^18^ Thus, the change in morphology from nanoflowers to nanocarambolas occurred after an increase in PVP concentration by a factor of 18. The second study found that using 0.06 mol/L oxalic acid produced nanorods, while 0.12 mol/L oxalic acid produced a mixture of nanobundles and nanocarambolas.^7^ Both of these studies concluded that the interaction of ligands with the surface of the nanocrystal during growth impacts the final morphology.^7,18,38^

To explore the mechanism by which the unusual nanocarambola morphology forms, we varied the duration of the reactions run in the presence of trifluoroacetic acid (see Supporting Information). At early reaction times (3 h), we observe nanorods with short rod-like protrusions growing perpendicular to the long axis. After 6 h, we observe fully formed nanocarambolas. We hypothesize that the short protrusions observed at early times expand along the long axis of the original nanorod, forming sheets that result in the 6-armed nanocarambola structure. Unlike other carboxylic acids used here, trifluoroacetic acid has two moieties that can engage in strong interactions with the nanocrystal surface: the carboxylate group and the fluorine atoms. Carboxylic acid groups are well known to bind to the surfaces of metal oxide nanoparticles.^39,40^ In the case of TiO_2_, trifluoroacetic acid ligands were found to have a similar structure-directing effect as the addition of HF—both additives resulted in the stabilization of {001} facets and the formation of nanosheets in the case of HF and a mixture of nanosheets and truncated octahedrons in the case of trifluoroacetic acid.^41^ The authors also observed that aliphatic carboxylic acids, such as oleic acid, form truncated octahedrons, indicating that the carboxylate group also stabilizes {001} facets but not to the same extent as fluoride ions. The authors attributed the intermediate structure-directing effect of trifluoroacetic acid to its partial degradation under solvothermal reaction conditions to form fluoride ions. We suspect that similar degradation of a fraction of the trifluoroacetic acid molecules may be responsible for the formation of VO_2_(B) nanocarambolas.

## Conclusions

Our work demonstrates the effects of the acidity of the reaction mixture on the crystal phase and morphology of vanadium dioxide nanocrystals obtained under solvothermal reaction conditions. Decreasing the p*K*_a_ and increasing the concentration of carboxylic acid ligands present in the reaction mixture while keeping the concentration of water constant favors the formation of VO_2_(A), whereas increased p*K*_a_ and decreased acid concentration favor the formation of VO_2_(B). Furthermore, increasing the concentration of the precursor relative to the concentration of water leads to VO_2_(A), whereas VO_2_(B) forms from reaction mixtures containing lower precursor-to-water ratios. These observations are consistent with our hypothesis that the rate of precursor hydrolysis relative to condensation controls which crystal phase nucleates. We identified VO(carboxylate)_2_ species as likely reaction intermediates formed prior to nucleation of the VO_2_ species. Finally, our results suggest that acidity alone likely does not have a strong impact on nanocrystal morphology. Instead, interactions involving substituents on the carboxylic acid ligands, such as intermolecular π–π interactions or interactions between fluoride ions formed upon degradation of −CF_3_ groups and the nanocrystal surface, may play a significant role in determining the morphology of VO_2_(B) nanocrystals. Overall, this work provides important insight into how carboxylic acids impact the crystal phase and morphology of VO_2_ nanocrystals.

## Experimental Methods

### General Considerations

Prior to the addition of water to the reaction mixtures, all manipulations were carried out in an MBraun inert atmosphere glovebox under a nitrogen atmosphere unless otherwise stated. Autoclave reactors and Teflon liners were pumped down in an evacuated antechamber overnight prior to use in the glovebox. Molecular sieves (3 Å, Fisher Scientific) were activated by heating at 200 °C under vacuum overnight. Toluene was dried over activated sieves (20% by weight) for at least 24 h, then purged with nitrogen for at least 1 h before being transferred via a cannula to a Schlenk flask. Toluene was then pumped into the glove box overnight in an evacuated antechamber and stored over activated 3 Å molecular sieves. VO(acac)_2_, stearic acid, decanoic acid, heptanoic acid, hexanoic acid, and butyric acid purchased from Sigma-Aldrich and lauric acid purchased from Millipore were pumped into the glovebox overnight in an evacuated antechamber before use. Benzoic acid and 4-nitrobenzoic acid purchased from Oakwood, acetic acid purchased from Fisher Scientific, and trifluoroacetic acid purchased from Sigma-Aldrich were used without further purification.

### Safety Considerations

Trifluoroacetic acid is both volatile and corrosive and should therefore be handled exclusively in a fume hood. Autoclave reactors become pressurized at elevated temperatures and should be handled with care. No attempts should be made to open autoclave reactors unless they are at room temperature.

### Synthesis of VO_2_ Nanocrystals in the Presence of Different Carboxylic Acids

A 25 mL Teflon-lined autoclave reactor was charged with 0.25 mmol (0.068 g) VO(acac)_2_ and 10 mL of toluene inside a nitrogen-filled glovebox. The autoclave reactor was sealed and removed from the glovebox. In ambient air, the autoclave reactor was opened, and the desired amount of water was added (3–20 mmol). A carboxylic acid (1 mmol) was added to the reaction mixture either before (stearic, lauric, decanoic, heptanoic, hexanoic, or butyric) or after (lauric, acetic, benzoic, 4-nitrobenzoic, or trifluoroacetic acid) removal from the glovebox. Tables S1 and S4 in the Supporting Information contain detailed lists of the masses and volumes of the various reagents used in these reactions. After the addition of water, the autoclave reactor was sealed again and heated in an oven at 200 °C for 24 h before it was removed and allowed to cool to room temperature. The black solid product was collected by centrifugation under ambient conditions and washed with ethanol. The product was rotovapped to dryness and stored in the glovebox under nitrogen.

### Synthesis of VO_2_ Nanocrystals with Various Concentrations of Precursor, Water, and Acid

A 25 mL Teflon-lined autoclave reactor was charged with the desired amount of VO(acac)_2_ (0.075–3 mmol) and 10 mL of toluene. The autoclave reactor was sealed and removed from the glovebox. In ambient air, the autoclave reactor was opened and the desired amounts of water (0.5–20 mmol) and trifluoroacetic acid (0.5–4 mmol) were added (see Table S3 in the Supporting Information for the details of each individual reaction reported here). The autoclave reactor was sealed again and heated in an oven at 200 °C for 24 h before it was removed from the oven and allowed to cool to room temperature. The black solid product was collected by centrifugation under ambient conditions and washed with ethanol. The product was rotovapped to dryness and stored in the glovebox under nitrogen. Vanadyl carboxylate products were collected and purified using procedures identical to those used for the vanadium dioxide nanocrystals.

### Nanocrystal Characterization

Powder X-ray diffraction measurements were performed using a Rigaku XtaLAB Synergy-S Dualflex single-crystal diffractometer operating in the powder collection mode using Cu Kα radiation. Nanocrystalline powders were affixed to a Nylon loop (0.1 mm inner diameter) with a light coating of viscous oil and mounted on a goniometer for data collection. An FEI Tecnai F20 G2 scanning transmission electron microscope operated at 200 kV and a Zeiss Auriga scanning electron microscope with an InLens detector operated at 5–20 kV were used to analyze the morphology of the VO_2_ nanocrystals. Single nanocrystals of VO(benzoate)_2_ and VO(4-nitrobenzoate)_2_ were structurally analyzed using a Rigaku XtaLAB Synergy-ED electron diffractometer equipped with a HyPix-ED detector (see Supporting Information for details).

## Supporting Information Available

Tables reporting the amounts of all reagents used in each reaction reported here, powder X-ray diffraction spectra, TEM, and SEM images of nanocrystalline products, and details of the characterization of VO(benzoate)_2_ and VO(4-nitrobenzoate)_2_ by single-crystal electron diffraction (PDF)
Crystallographic data for VO(benozate)_2_ (CIF)
Crystallographic data for VO(4-nitrobenzoate)_2_ (CIF)

## Supplementary Material

ng3c00014_si_001.pdf

ng3c00014_si_002.cif

ng3c00014_si_003.cif
