# Supplementary material for: Acidity of Carboxylic Acid Ligands Influences the Formation of VO2(A) and VO2(B) Nanocrystals under Solvothermal Conditions
Source: ACS Nanosci Au. 2023 Jun 22;3(5):381–8. doi: 10.1021/acsnanoscienceau.3c00014 (PMC10588437; doi:10.1021/acsnanoscienceau.3c00014)
Supplement: Supplementary file 1 — ng3c00014_si_001.pdf [file ng3c00014_si_001.pdf]

Supporting Information for

Acidity of Carboxylic Acid Ligands Influences Formation of VO_2(A) and VO_2(B) Nanocrystals Under Solvothermal Conditions

Brittney A. Beidelman, Xiaotian Zhang, Ellen M. Matson, and Kathryn E. Knowles*

Department of Chemistry, University of Rochester, Rochester, NY 14627, USA

*Corresponding Author Email: kknowles@ur.rochester.edu

Supporting Information Table of Contents:

Table S1. Experimental Details of Nanocrystal Reactions Shown in Figure 1 of Main Text.................... S2

Figures S1-S6. Powder X-ray diffraction spectra corresponding to reactions in Table S1....................S3-5

Experimental Details of Single Crystal Electron Diffraction measurements..S6

Table S2. Crystal data summary for structures VO(benzoate)$_2$ and VO(4-nitrobenzoate)$_2$.......................S7

Figure S7. Powder X-ray diffraction and crystal packing structures of VO(OOCR)$_2$ species...................S8

Figure S9. Powder X-ray diffraction spectra of products obtained in the absence of water and presence of lauric or benzoic acid..S8

Table S3. Experimental Details of Nanocrystal Reactions Shown in Figure 3 of Main Text..................S9

Figures S9-12. Powder X-ray diffraction spectra corresponding to reactions in Table S3................S10-S11

Figure S13. Powder X-ray diffraction spectra of products collected after various reaction times............S12

Table S4. Experimental Details of Nanocrystal Reactions Run with Carboxylic Acids of Various Carbon Chain Lengths...S12

Figure S14. Powder X-ray Diffraction and TEM images corresponding to reactions in Table S4...........S13

Figure S15. TEM images of VO_2(B) nanocrystals obtained after various reaction times in the presence of trifluoroacetic acid...S13

Table S1. Experimental Details of Nanocrystal Reactions Shown in Figure 1 of Main Text[a]

Acid Used	$VO(acac)_2$ (g)	$VO(acac)_2$ (mmol)	Carboxylic Acid (g)	Carboxylic Acid (mmol)	Water (g)	Water (mmol)	Product
No Acid	0.068	0.25	none	none	0.009	0.5	VO_2(A)
No Acid	0.068	0.25	none	none	0.018	1	VO_2(A)
No Acid	0.068	0.25	none	none	0.054	3	VO_2(B)
No Acid	0.068	0.25	none	none	0.090	5	VO_2(B)
No Acid	0.068	0.25	none	none	0.18	10	VO_2(B)
No Acid	0.068	0.25	none	none	0.27	15	VO_2(B)
No Acid	0.068	0.25	none	none	0.36	20	VO_2(B)
Lauric	0.068	0.25	0.200	1	0.009	0.5	VO_2(A)
Lauric	0.068	0.25	0.200	1	0.018	1	VO_2(A)
Lauric	0.068	0.25	0.200	1	0.054	3	VO_2(A)/VO_2(B)[b]
Lauric	0.068	0.25	0.200	1	0.090	5	VO_2(B)
Lauric	0.068	0.25	0.200	1	0.18	10	VO_2(B)
Lauric	0.068	0.25	0.200	1	0.54	30	VO_2(B)
Acetic	0.068	0.25	0.060	1	0.009	0.5	VO(acetate)$_2$
Acetic	0.068	0.25	0.060	1	0.018	1	VO(acetate)$_2$
Acetic	0.068	0.25	0.060	1	0.045	2.5	VO_2(A)/VO_2(B)[b]
Acetic	0.068	0.25	0.060	1	0.072	4	VO_2(A)/VO_2(B)[b]
Acetic	0.068	0.25	0.060	1	0.09	5	VO_2(B)
Acetic	0.068	0.25	0.060	1	0.18	10	VO_2(B)
Acetic	0.068	0.25	0.060	1	0.27	15	VO_2(B)
Acetic	0.068	0.25	0.060	1	0.36	20	VO_2(B)
Benzoic	0.068	0.25	0.122	1	0.009	0.5	VO(benzoate)$_2$
Benzoic	0.068	0.25	0.122	1	0.018	1	VO(benzoate)$_2$
Benzoic	0.068	0.25	0.122	1	0.054	3	VO_2(A)
Benzoic	0.068	0.25	0.122	1	0.09	5	VO_2(B)
Benzoic	0.068	0.25	0.122	1	0.18	19	VO_2(B)
Benzoic	0.068	0.25	0.122	1	0.27	15	VO_2(B)
Benzoic	0.068	0.25	0.122	1	0.36	20	VO_2(B)
4-Nitrobenzoic	0.068	0.25	0.167	1	0.009	0.5	VO(4-nitrobenzoate)$_2$
4-Nitrobenzoic	0.068	0.25	0.167	1	0.018	1	VO(4-nitrobenzoate)$_2$
4-Nitrobenzoic	0.068	0.25	0.167	1	0.054	3	VO(4-nitrobenzoate)$_2$ /VO_2(B)[c]
4-Nitrobenzoic	0.068	0.25	0.167	1	0.09	5	VO_2(B)
4-Nitrobenzoic	0.068	0.25	0.167	1	0.18	10	VO_2(B)
4-Nitrobenzoic	0.068	0.25	0.167	1	0.27	15	VO_2(B)
4-Nitrobenzoic	0.068	0.25	0.167	1	0.36	20	VO_2(B)
Trifluoroacetic	0.068	0.25	0.114	1	0.054	3	VO_2(A)
Trifluoroacetic	0.068	0.25	0.114	1	0.09	5	VO_2(A)
Trifluoroacetic	0.068	0.25	0.114	1	0.19	10	VO_2(A)
Trifluoroacetic	0.068	0.25	0.114	1	0.216	12	VO_2(A)
Trifluoroacetic	0.068	0.25	0.114	1	0.27	15	VO_2(B)
Trifluoroacetic	0.068	0.25	0.114	1	0.324	18	VO_2(B)
Trifluoroacetic	0.068	0.25	0.114	1	0.36	20	VO_2(B)

[a]Corresponding powder XRD spectra shown in Figures S1-S6.

[b]Cannot distinguish between VO_2(A) and VO_2(B) in powder XRD pattern of these products

[c]Powder XRD pattern shows features consistent with both VO(4-nitrobenzoate)$_2$ and VO_2(B)

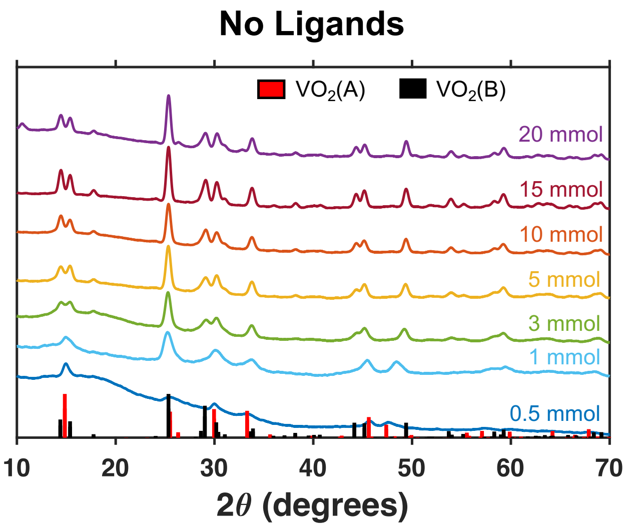

Figure S1. Powder X-ray diffraction spectra of nanocrystals obtained from solvothermal reactions of 0.25 mmol of $VO(acac)_2$ in toluene with 0.5-20 mmol of water and no carboxylic acids present.

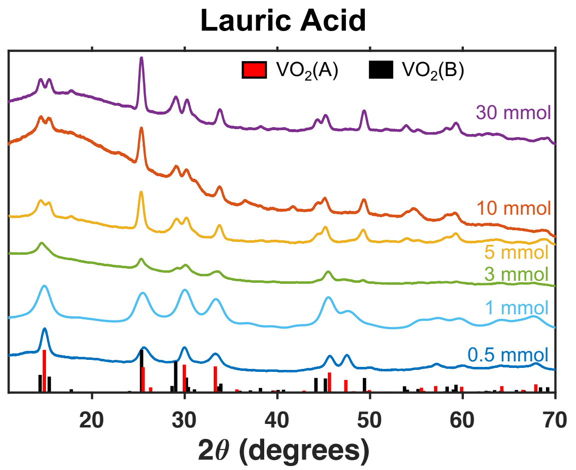

Figure S2. Powder X-ray diffraction spectra of nanocrystals obtained from solvothermal reactions of 0.25 mmol $VO(acac)_2$ in toluene with 0.5-20mmol of water and 1 mmol of lauric acid.

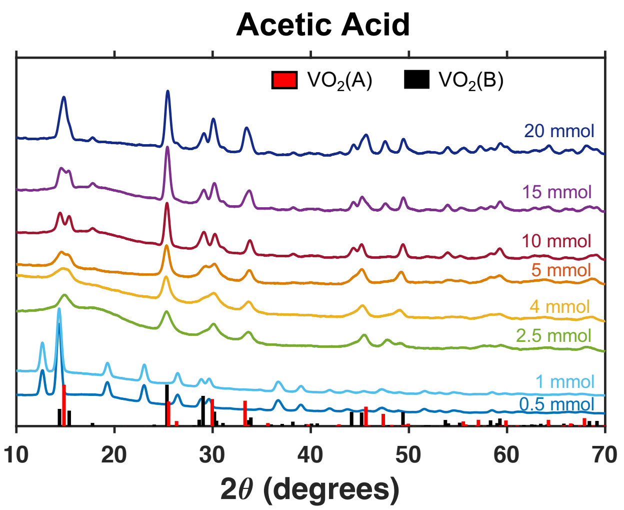

Figure S3. Powder X-ray diffraction spectra of nanocrystals obtained from solvothermal reactions of 0.25 mmol VO(acac)$_2$ in toluene with 0.5-20mmol of water and 1 mmol of acetic acid.

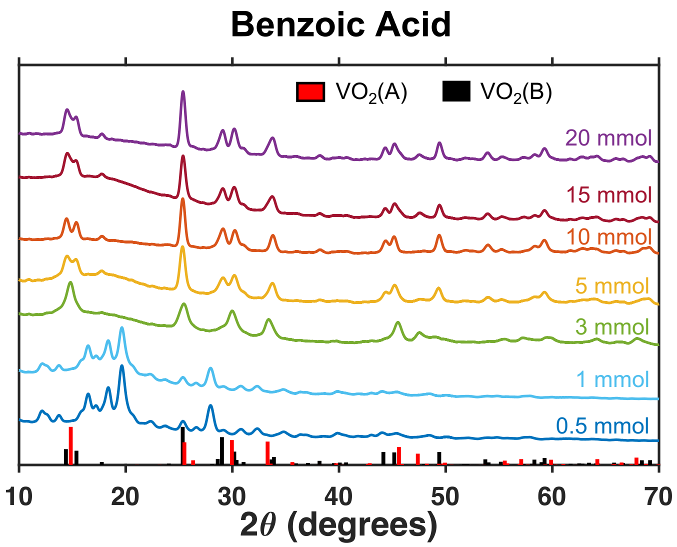

Figure S4. Powder X-ray diffraction spectra of nanocrystals obtained from solvothermal reactions of 0.25 mmol VO(acac)$_2$ in toluene with 0.5-20mmol of water and 1 mmol of benzoic acid.

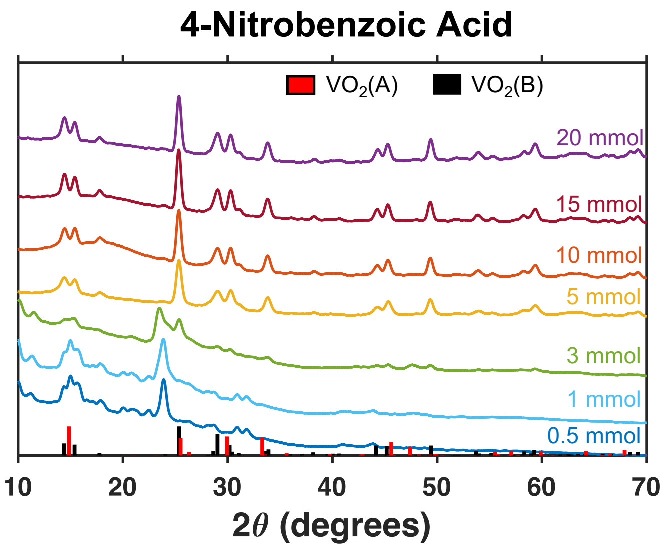

Figure S5. Powder X-ray diffraction spectra of nanocrystals obtained from solvothermal reactions of 0.25 mmol $VO(acac)_2$ in toluene with 0.5-20mmol of water and 1 mmol of 4-nitrobenzoic acid.

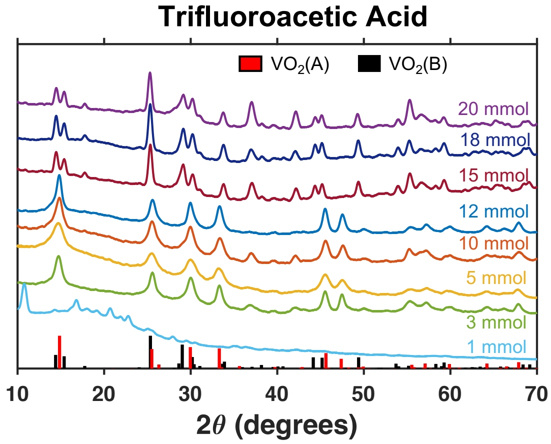

Figure S6. Powder X-ray diffraction spectra of nanocrystals obtained from solvothermal reactions of 0.25 mmol $VO(acac)_2$ in toluene with 1-20mmol of water and 1 mmol of trifluoroacetic acid.

Single Crystal Electron Diffraction of VO(benzoate)$_2$ and VO(4-nitrobenzoate)$_2$.

Single nanocrystals of VO(benzoate)$_2$ and VO(4-nitrobenzoate)$_2$ were structurally analyzed using single crystal electron diffraction (MicroED). Multiple crystallites were placed onto a screening tray and examined under vacuum at room temperature on a Rigaku XtaLAB Synergy-ED diffractometer equipped with a HyPix-ED detector. Intensity data were generated by electron diffraction (λ = 0.0251 Å) from four (VO(benzoate)$_2$) and three (VO(4-nitrobenzoate)$_2$) crystallites, from which the best sets for each sample were merged.[71]

Structures were solved using *SHELXT*[72] and refined using *SHELXL*.[73] Space groups were determined based on systematic absences (VO(benzoate)$_2$) or intensity statistics (VO(4-nitrobenzoate)$_2$). Most or all non-hydrogen atoms were assigned from the solution. Full-matrix least squares / difference Fourier cycles were performed which located any remaining non-hydrogen atoms. All non-hydrogen atoms were refined with anisotropic displacement parameters. All hydrogen atoms were placed in ideal positions and refined as riding atoms with relative isotropic displacement parameters. Full matrix least squares refinements on F^2 were run to convergence.

Structure manipulation and figure generation were performed using *Olex2*.[74] See Table S1 in Appendix A for additional experimental details and results.

Table S2. Crystal data summary for structures VO(benzoate)$_2$ and VO(4-nitrobenzoate)$_2$.

	VO(benzoate)$_2$	VO(4-nitrobenzoate)$_2$
formula	$C_{14}H_{10}O_5V$	$C_{14}H_8N_2O_9V$
formula weight	309.17	399.16
T (K)	293(2)	293(2)
wavelength (Å)	0.0251	0.0251
crystal system	monoclinic	triclinic
space group	$P2_1/c$	P-1
a (Å)	10.1(4)	6.9008(6)
b (Å)	6.95(15)	16.1046(17)
c (Å)	21.0(3)	18.4938(17)
α (deg)	90	84.106(8)
β (deg)	91.9(2)	81.289(8)
γ (deg)	90	86.829(8)
V (Å^3)	1478(73)	2019.2(3)
Z	4	4
ρ_{calcd} (g cm^{-3})	1.390	1.313
color, shape	yellow, needle	brown, needle
reflections collected	8799	25995
reflections independent	2789	7128
R_{int}^{a}	0.1080	0.1573
reflections observed	2515	4515
number of parameters	170	470
completeness to 0.80 Å(%)	90.4	86.3
extinction parameter^b	5054(12)	253(9)
GOF^c on F^2	2.754	2.357
$R1$ [$I > 2\sigma(I)$]^d	0.2413	0.2226
$wR2^e$	0.6082	0.4692

$^a R_{int} = \Sigma |F_o^2 - \langle F_o^2 \rangle| / \Sigma F_o^2$.
^{b}Extinction parameter x: Fc is multiplied by $k[1 + 0.001Fc^2\lambda^3x / \sin(2\theta)]^{-1/4}$, where k is the overall scale factor.
^{c}GOF = $S = [\Sigma w(F_o^2 - F_c^2)^2 / (m - n)]^{1/2}$, where $w = 1 / [\sigma^2(F_o^2) + (aP)^2 + bP]$, $P = 1/3 \max(0, F_o^2) + 2/3F_c^2$, m = number of independent reflections, and n = number of parameters.
$^d R1 = \Sigma ||F_o| - |F_c|| / \Sigma |F_o|$.
$^e wR2 = [\Sigma w(F_o^2 - F_c^2)^2 / \Sigma wF_o^2]^{1/2}$.

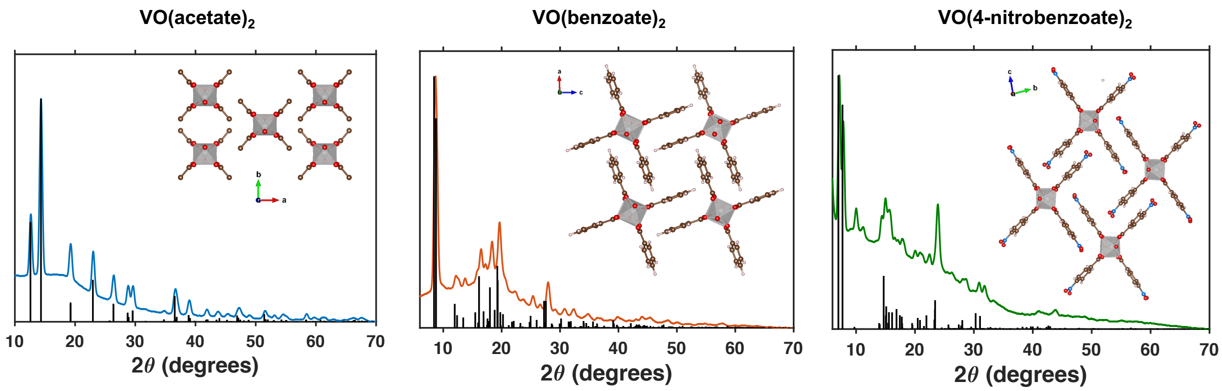

Figure S7. Powder X-ray diffraction spectra of products isolated from the solvothermal reaction of $VO(acac)_2$ with 4 equivalents of water and 4 equivalents of acetic acid (left), benzoic acid (middle) and nitrobenzoic acid (right) (reproduced from Figure 2 in main text). These patterns are consistent with the powder XRD patterns corresponding to $VO(acetate)_2$, $VO(benzoate)_2$, and $VO(4\text{-nitrobenzoate})_2$, respectively (shown as vertical black bars in each figure). The powder XRD pattern for $VO(acetate)_2$ was obtained from reference and the powder XRD patterns for $VO(benzoate)_2$ and $VO(4\text{-nitrobenzoate})_2$ were derived from the single crystal electron diffraction structures reported herein. (inset) End-on views of the one-dimensional chains of edge-sharing VO_6 octahedra in each carboxylate structure showing how these chains pack in the solid state. These structures illustrate the p-p stacking interactions between the aryl rings in $VO(benzoate)_2$ and $VO(4\text{-nitrobenzoaate})_2$.

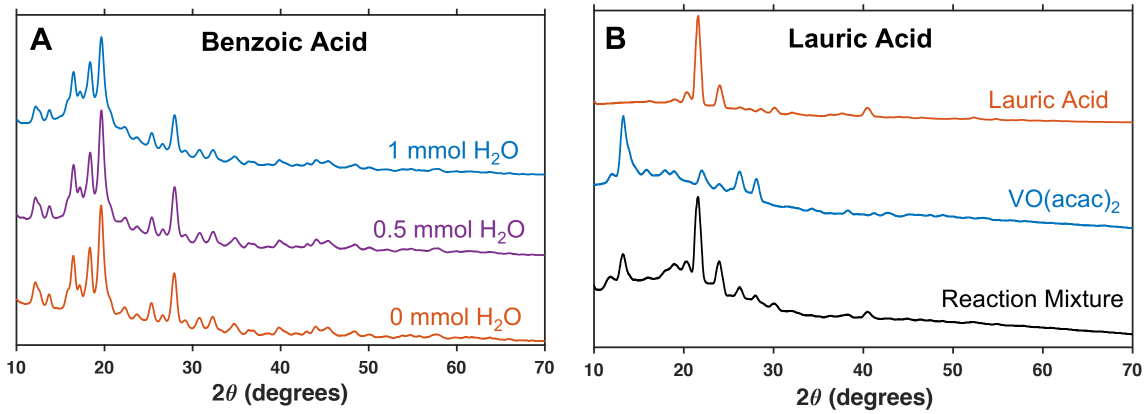

Figure S8. A) Powder X-ray diffraction spectra of products obtained from reactions of 0.25 mmol of $VO(acac)_2$ in toluene with 0-1 mmol of water and 1 mmol of benzoic acid. All reactions produce $VO(benzoate)_2$. **B)** Powder X-ray diffraction spectra of lauric acid (orange), $VO(acac)_2$ (blue) and the reaction mixture obtained from solvothermal reaction of 0.25 mmol of $VO(acac)_2$ and 1 mmol of lauric acid in toluene in the absence of water (black). The spectrum of the reaction mixture is consistent with the presence of both $VO(acac)_2$ and lauric acid and indicates that no reaction occurs under these conditions.

Table S3. Experimental Details of Nanocrystal Reactions Shown in Figure 3 of Main Text[a]

$VO(acac)_2$ (equiv)	$VO(acac)_2$ (mmol)	$VO(acac)_2$ (g)	TFA[b] (equiv)	TFA[b] (mmol)	TFA[b] (mL)	Water (equiv)	Water (mmol)	Water (g)	Product
1	0.25	0.068	4	1	0.076	12	3	0.054	VO_2(A)
1	0.25	0.068	4	1	0.076	20	5	0.09	VO_2(A)
1	0.25	0.068	4	1	0.076	40	10	0.18	VO_2(A)
1	0.25	0.068	4	1	0.076	48	12	0.216	VO_2(A)
1	0.25	0.068	4	1	0.076	60	15	0.27	VO_2(B)
1	0.25	0.068	4	1	0.076	72	18	0.324	VO_2(B)
1	0.25	0.068	4	1	0.076	80	20	0.36	VO_2(B)
1	0.25	0.068	4	1	0.076	120	30	0.54	VO_2(B)
1	0.25	0.068	4	1	0.076	160	40	0.72	VO_2(B)
1	0.25	0.068	4	1	0.076	200	50	0.9	VO_2(B)
4	1	0.272	4	1	0.076	12	3	0.054	VO_2(A)
4	1	0.272	4	1	0.076	20	5	0.09	VO_2(A)
4	1	0.272	4	1	0.076	40	10	0.18	VO_2(A)
4	1	0.272	4	1	0.076	60	15	0.27	VO_2(A)
4	1	0.272	4	1	0.076	80	20	0.36	VO_2(A)
4	1	0.272	4	1	0.076	120	30	0.54	VO_2(B)
4	1	0.272	4	1	0.076	160	40	0.72	VO_2(B)
4	1	0.272	4	1	0.076	200	50	0.9	VO_2(B)
0.5	0.125	0.034	4	1	0.076	60	15	0.27	VO_2(B)
0.8	0.2	0.054	4	1	0.076	60	15	0.27	VO_2(B)
1	0.25	0.068	4	1	0.076	60	15	0.27	VO_2(B)
2	0.5	0.136	4	1	0.076	60	15	0.27	VO_2(A)/ VO_2(B)[c]
4	1	0.272	4	1	0.076	60	15	0.27	VO_2(A)
8	2	0.544	4	1	0.076	60	15	0.27	VO_2(A)
12	3	0.816	4	1	0.076	60	15	0.27	VO_2(A)
1	0.25	0.068	2	0.5	0.038	60	15	0.27	VO_2(B)
1	0.25	0.068	3	0.75	0.057	60	15	0.27	VO_2(B)
1	0.25	0.068	4	1	0.076	60	15	0.27	VO_2(B)
1	0.25	0.068	8	2	0.152	60	15	0.27	VO_2(B)
1	0.25	0.068	10	2.5	0.19	60	15	0.27	VO_2(A)
1	0.25	0.068	12	3	0.228	60	15	0.27	VO_2(A)
1	0.25	0.068	16	4	0.304	60	15	0.27	VO_2(A)

[a]Corresponding powder XRD spectra shown in Figures S9-S12

[b]TFA = Trifluoroacetic acid

[c]Cannot distinguish between VO_2(A) and VO_2(B) in powder XRD pattern of this product

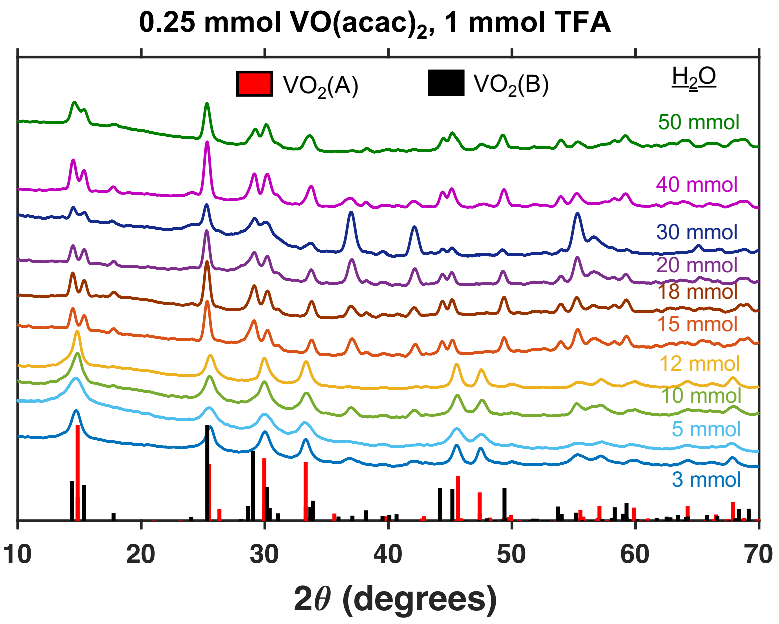

Figure S9. Powder X-ray diffraction spectra of nanocrystals obtained from solvothermal reactions of 0.25 mmol $VO(acac)_2$ in toluene with 1 mmol trifluoroacetic acid and 3-50 mmol of water.

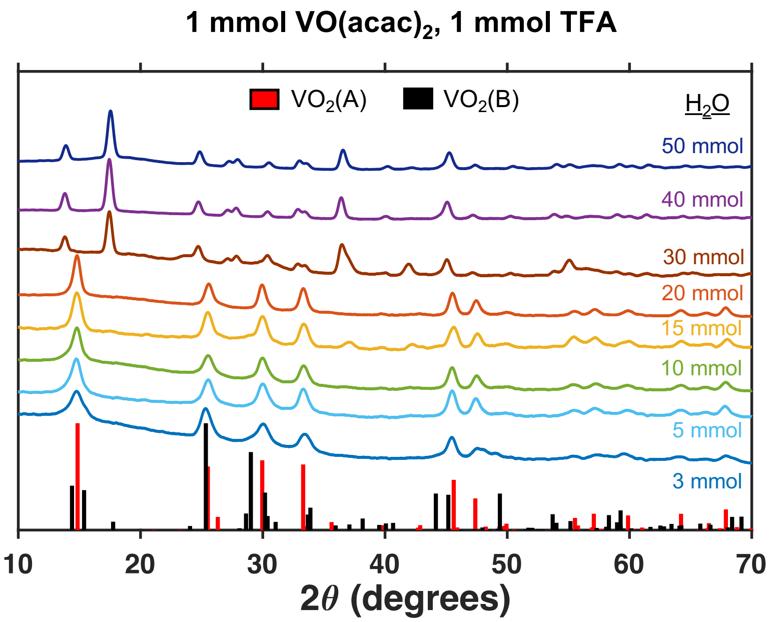

Figure S10. Powder X-ray diffraction spectra of nanocrystals obtained from solvothermal reactions of 1 mmol $VO(acac)_2$ in toluene with 1 mmol trifluoroacetic acid and 3-50 mmol of water.

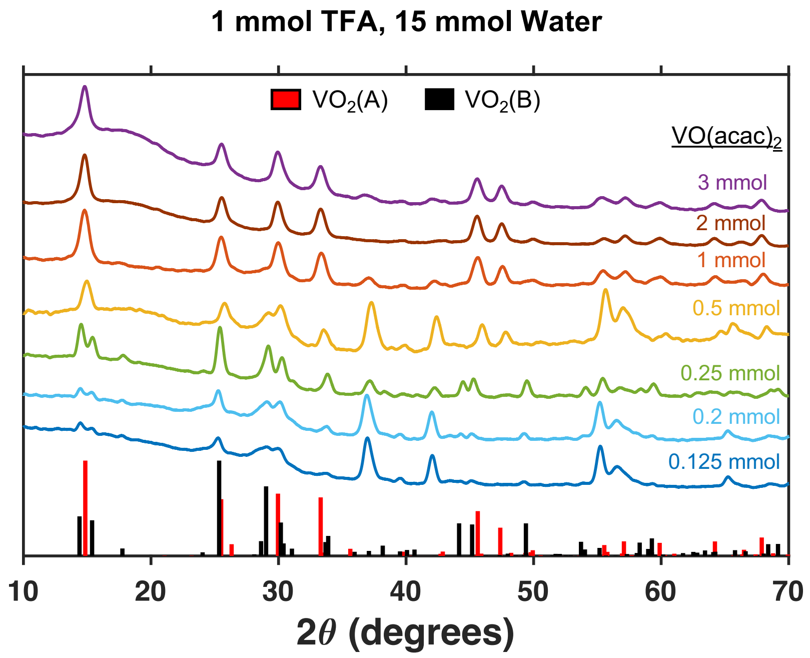

Figure S11. Powder X-ray diffraction spectra of nanocrystals obtained from solvothermal reactions of 0.125-3 mmol $VO(acac)_2$ in toluene with 1 mmol trifluoroacetic acid and 15 mmol of water.

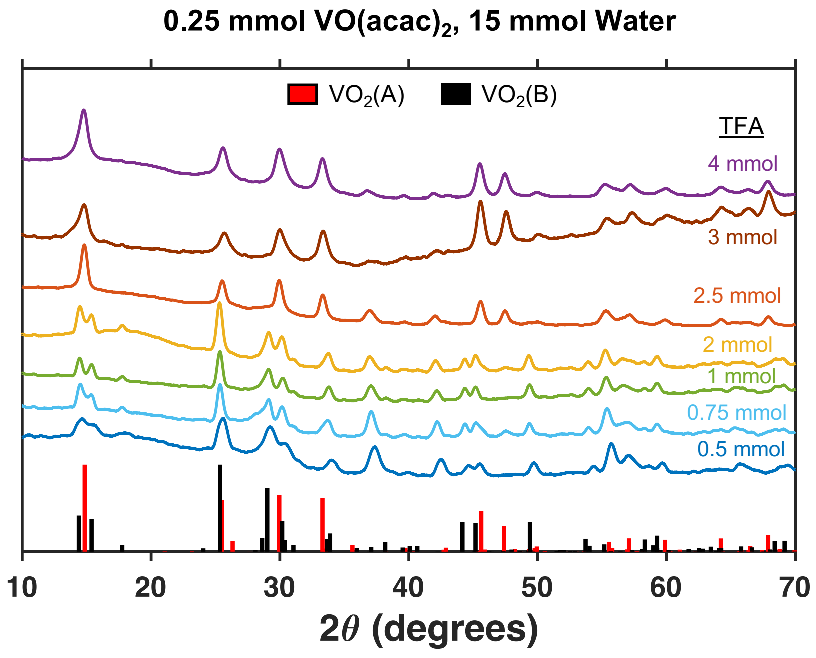

Figure S12. Powder X-ray diffraction spectra of nanocrystals obtained from solvothermal reactions of 0.25 mmol $VO(acac)_2$ in toluene with 15 mmol water and 0.5-4 mmol of trifluoroacetic acid.

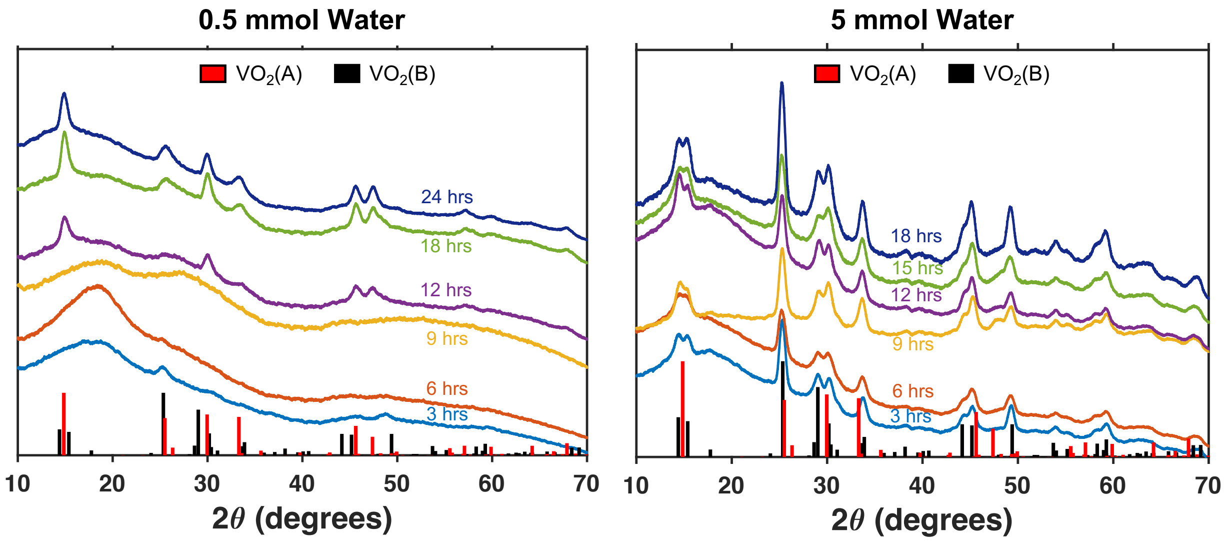

Figure S13. Powder X-ray diffraction spectra of products collected after various reaction times at 200 C from reaction mixtures containing 0.25 mmol $VO(acac)_2$, 1 mmol of lauric acid, and 0.5 (left) or 5 (right) mmol of water.

Table S4. Experimental Details of Nanocrystal Reactions Run with Carboxylic Acids of Various Carbon Chain Lengths

Acid Used	$VO(acac)_2$ (g)	$VO(acac)_2$ (mmol)	Carboxylic Acid (g)	Carboxylic Acid (mmol)	Water (g)	Water (mmol)	Product
Stearic	0.068	0.25	0.284	1	0.09	5	VO_2(B)
Lauric	0.068	0.25	0.200	1	0.09	5	VO_2(B)
Decanoic	0.068	0.25	0.172	1	0.09	5	VO_2(B)
Heptanoic	0.068	0.25	0.130	1	0.09	5	VO_2(B)
Hexanoic	0.068	0.25	0.116	1	0.09	5	VO_2(B)
Butyric	0.068	0.25	0.088	1	0.09	5	VO_2(B)
None	0.068	0.25	none	1	0.09	5	VO_2(B)

[a]Corresponding powder XRD spectra shown in Figure S14.

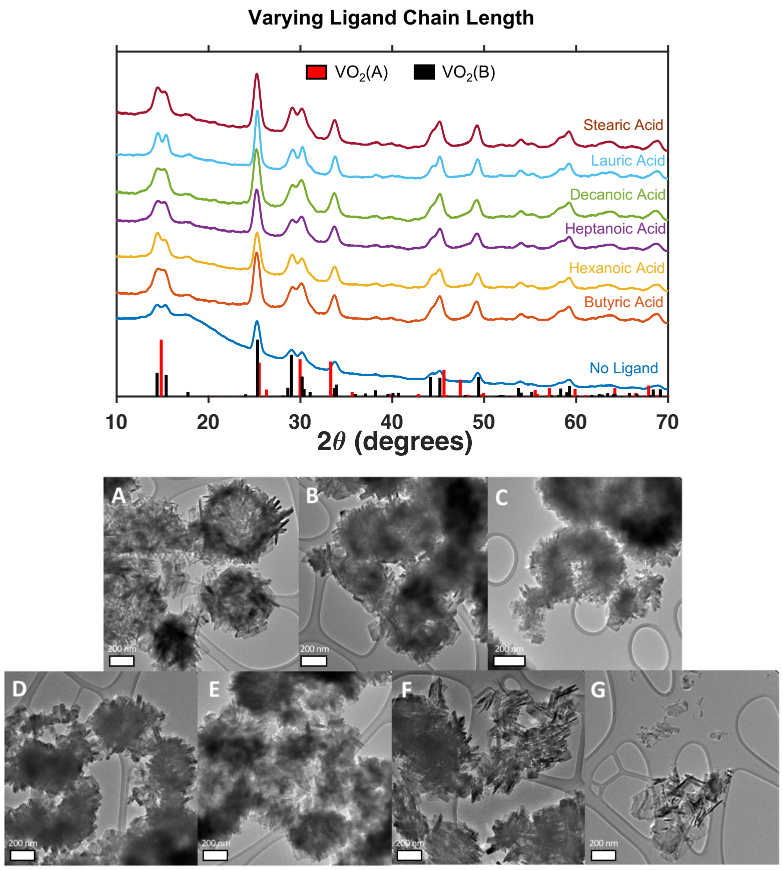

Figure S14. (*Top*) Powder X-ray diffraction spectra of nanocrystals obtained from solvothermal reactions of 0.25 mmol VO(acac)$_2$ in toluene with 5 mmol of water and 1 mmol of carboxylic acids of various chain lengths. All reactions yielded VO_2(B) (JCPDS 01-081-2392). (*Bottom*) Transmission electron microscopy images of VO_2(B) nanocrystals synthesized with (**A**) No Ligand, (**B**) Butyric acid (4 carbons), (**C**) Hexanoic acid (6 carbons), (D) Heptanoic acid (7 carbons), (**E**) Decanoic acid (10 carbons), (**F**) Lauric acid (12 carbons), (**G**) Stearic acid (18 carbons). All scale bars are 200 nm

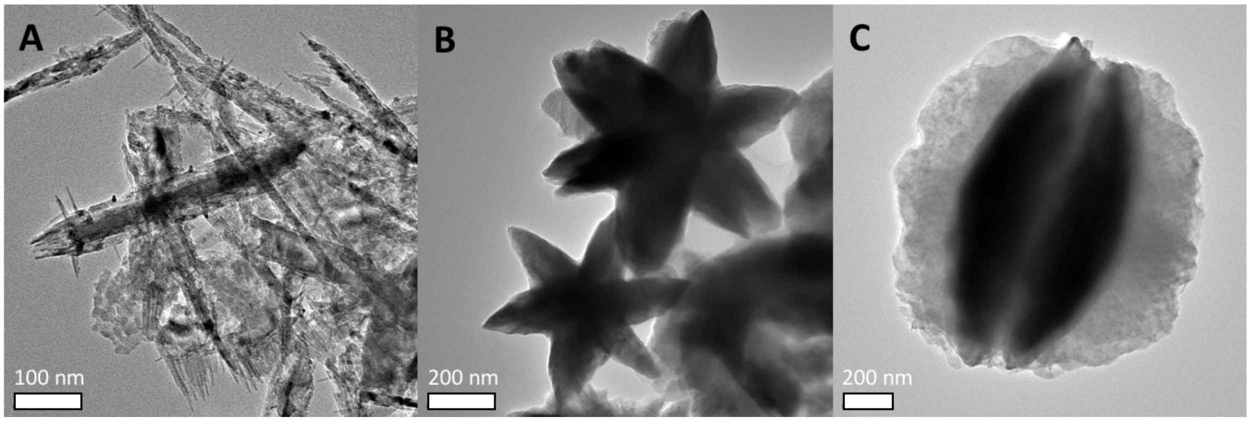

Figure S15. TEM images of VO_2(B) nanocrystals obtained from reactions containing 1 equivalent (0.25 mmol) VO(acac)$_2$, 4 equivalents (1 mmol) of trifluoroacetic acid, 60 equivalents (15 mmol) water, and 10mL toluene at 3 hours (**A**), 6 hours (**B**), and 9 hours (**C**).
